# Substrate Stiffness Regulates Filopodial Activities in Lung Cancer Cells

**DOI:** 10.1371/journal.pone.0089767

**Published:** 2014-02-27

**Authors:** Yu-Ren Liou, Wen Torng, Yu-Chiu Kao, Kung-Bin Sung, Chau-Hwang Lee, Po-Ling Kuo

**Affiliations:** 1 Graduate Institute of Biomedical Electronics and Bioinformatics, National Taiwan University, Taipei, Taiwan; 2 Department of Electrical Engineering, National Taiwan University, Taipei, Taiwan; 3 Research Center for Applied Sciences, Academia Sinica, Taipei, Taiwan; 4 Institute of Biophotonics, National Yang-Ming University, Taipei, Taiwan; 5 Department of Rehabilitation, National Taiwan University Hospital, Taipei, Taiwan; China Medical University, Taiwan

## Abstract

Microenvironment stiffening plays a crucial role in tumorigenesis. While filopodia are generally thought to be one of the cellular mechanosensors for probing environmental stiffness, the effects of environmental stiffness on filopodial activities of cancer cells remain unclear. In this work, we investigated the filopodial activities of human lung adenocarcinoma cells CL1-5 cultured on substrates of tunable stiffness using a novel platform. The platform consists of an optical system called structured illumination nano-profilometry, which allows time-lapsed visualization of filopodial activities without fluorescence labeling. The culturing substrates were composed of polyvinyl chloride mixed with an environmentally friendly plasticizer to yield Young's modulus ranging from 20 to 60 kPa. Cell viability studies showed that the viability of cells cultured on the substrates was similar to those cultured on commonly used elastomers such as polydimethylsiloxane. Time-lapsed live cell images were acquired and the filopodial activities in response to substrates with varying degrees of stiffness were analyzed. Statistical analyses revealed that lung cancer cells cultured on softer substrates appeared to have longer filopodia, higher filopodial densities with respect to the cellular perimeter, and slower filopodial retraction rates. Nonetheless, the temporal analysis of filopodial activities revealed that whether a filopodium decides to extend or retract is purely a stochastic process without dependency on substrate stiffness. The discrepancy of the filopodial activities between lung cancer cells cultured on substrates with different degrees of stiffness vanished when the myosin II activities were inhibited by treating the cells with blebbistatin, which suggests that the filopodial activities are closely modulated by the adhesion strength of the cells. Our data quantitatively relate filopodial activities of lung cancer cells with environmental stiffness and should shed light on the understanding and treatment of cancer progression and metastasis.

## Introduction

Microenvironment stiffness plays a crucial role in cancer development and progression. Stiffening of extracellular matrix resulting from increased collagen crosslinking occurs during tumorigenesis [1], [2]. The matrix stiffening affects cell motility, directs the migration of cancer cells, and may further be related to organ-specific metastasis [3]. Stiff matrix promotes the stability of cell focal adhesion, which enhances intracellular growth factor signaling and in turn increases tumor cell transformation and growth [2], [4]. For example, it was shown recently that several lung cancer cell lines grew better on stiffer substrates [5], and that reduction of matrix stifferening by inhibiting the lysyl oxidase-mediated collagen crosslinking impeded tumor progression [6]. Understanding how cancer cells sense and respond to environmental stiffness should provide valuable insights into the intricacies of cancer progression and assist in the improvement of treatment strategies.

Filopodia, finger-like protrusions at cell edges, are generally observed in highly metastatic cancer cells, such as CL1-5, a highly invasive human lung adenocarcinoma cells [7], [8]. The unique morphology and highly dynamic activities of filopodia make them intrinsically suitable organelles for probing environmental stiffness. Filopodia typically extend and retract within a time scale of tens of seconds, while their long length and high surface-to-volume ratio allow an intimate interaction with the microenvironment. Filopodial retraction involves the retrograde flow of F-actin primarily driven by myosin II contraction [9], while the myosin activities are positively correlated with substrate stiffness [4], [10]. Thus it is thought that filopodia may act as cellular mechanosensors by probing environmental stiffness at retraction. Recently, the substrate stiffness-sensitive dynamics of filopodia was demonstrated in neural growth cones and explained by a stochastic model based on the “motor-clutch” hypothesis [11], [12]. The model predicts that the myosin-driven retrograde flow rate of F-actin increases and the filopodial traction force decreases with increasing substrate stiffness. The experimental results confirmed that the filopodia detached from the substrate more frequently with higher substrate stiffness. If these predictions and observations can be generalized to cancer cells, one may expect that the overall filopodial activities of a cancer cell such as distribution of filopodial length and density would also be regulated by substrate stiffness. This is important since the presence and activities of filopodia in cancer cells are thought to be correlated with the cancer cell's ability to home to blood vessels and invade tissue [7], [13]–[15].

However, the effects of substrate stiffness on the filopodial activities of cancer cells remain unclear due to several technique limitations. The diameters of filopodia typically range from one to three hundred nanometers, which are at the margin of the resolution limit of conventional optical microscopy. Consequently, most live cell images regarding filopodial activities were taken from fluorescent protein-actin-transfected embryonic neurons, which have large filopodia at growth cones. However, the enhanced expression of the transfected fluorescent protein-actin complex may alter filopodial activities, while the phototoxicity brought by the excitation light may affect cell activities and change the dynamics of filopodia [16], [17].

In this work, we investigated the effects of substrate stiffness on the filopodial activities of the lung cancer cells CL1–5 using a newly developed imaging technique called structured-illumination nano-profilometry (SINAP), which utilizes topographical sensitivity to enhance the image contrast of a filopodium on flat substrates and allows label-free, time-lapsed visualization of filopodial activities at a frame rate up to 0.2 Hz [18], [19]. To improve the image contrast between filopodia and the surrounding medium, polyvinyl chloride (PVC) based materials with high refractive indices and tunable stiffness were adapted for cell culture. The biocompatibility of the PVC-based substrates was evaluated using MTT assay [20]. We quantified the filopodial activities of individual cells by measuring the filopodial density (i.e., the number of filopodia per unit length of the cellular peripheries), the average filopodial length, the filopodial extension and retraction rates, and the extension/retraction probability of individual filopodia, which is defined as the fraction of time that a filopodium spent for extension/retraction. To determine whether the effect of substrate stiffness on the filopodial activities is dependent on myosin II activities, we also measured the change of filopodial activities when cells cultured on substrates with different degrees of stiffness were treated with blebbistatin, a myosin II inhibitor.

## Results

### Characterization of the PVC-based substrates

The culture substrates were made of a mixture of PVC and an environmentally friendly, carboxylate type plasticizer, di(isononyl)cyclohexane-1, 2-dicardoxylate (DINCH). The stiffness of the mixture was tuned by adjusting the ratio of PVC to the plasticizer, while larger ratios resulted in stiffer composites. The Young's moduli of the PVC composites, as measured by applying sequential compression to the bulk materials, were 20.2±2.5 kPa (*n* = 6), 35.7±0 kPa (*n* = 1), and 61.1±12.9 kPa (*n* = 6) for PVC 1∶1, PVC 2∶1, and PVC 3∶1 respectively. Here the PVC 1∶1, 2∶1, and 3∶1 refer to the PVC composites with ratios of PVC to the plasticizer being 1∶1, 2∶1, and 3∶1 respectively. These data indicate that the stiffness of the PVC composites is within the range of most tissues in malignant conditions [21].

The working principle of SINAP requires that the culture substrate has a different refractive index from that of cells to improve the signal to noise ratio of the images. The refractive indices of the PVC composites were determined using the novel digital holographic microtomography as recently described [22]. The measured refractive indices of the PVC and the plasticizer were 1.53–1.57 and 1.47, respectively. Thus the resultant refractive indices of the PVC composite varied from 1.47 to 1.53, which is very close to that of glass (∼1.5) and higher than that of cells (∼1.36) [23].

**Figure 1:**
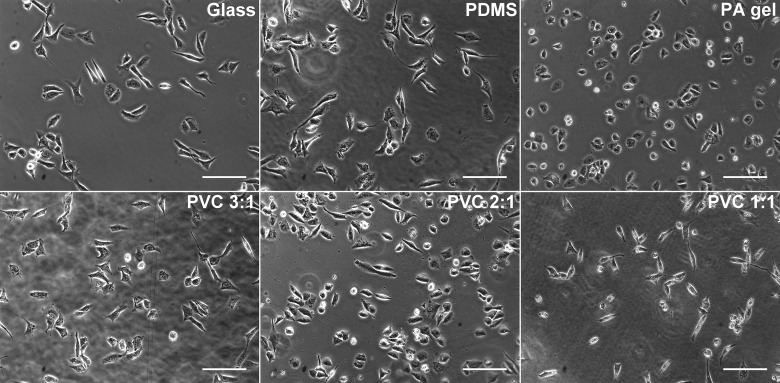
Phase contrast images of CL1–5 cancer cells grown on the surfaces of commonly used compliant substrates and glass coveslips. The PVC 1∶1, 2∶1, and 3∶1 refer to the PVC composites with ratios of PVC to the plasticizer being 1∶1, 2∶1, and 3∶1 respectively; PA and PDMS are abbreviated for polyacrylamide and poly(dimethyl) siloxane respectively. Scale bar  = 100 µm.

**Figure 2:**
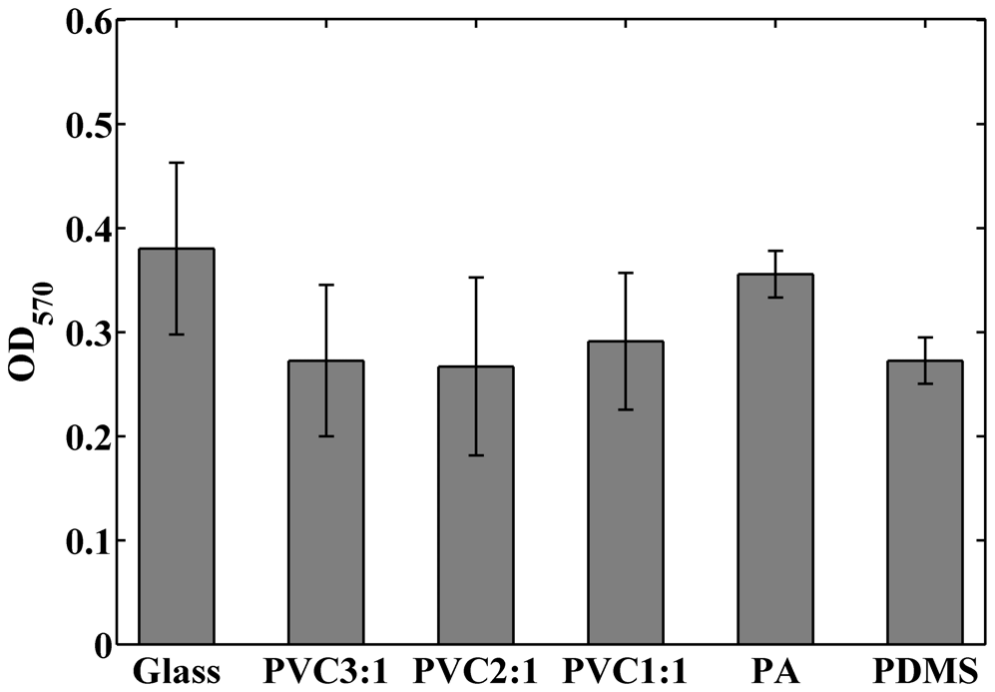
The optical densities of formazan dye in cells grown on various substrates. The dye has a purple color and was quantified by the optical absorbance at 570(*n* = 8, 5, 4, 4, 3, and 3 for the glass, PVC 3∶1, PVC 2∶1, PVC 1∶1, PA gel, and PDMS substrate respectively).

Cell viability test was used to determine whether the biocompatibility of the PVC composites is compatible with other compliant substrates commonly used for cell culture. We conducted MTT assays for human lung adenocarcinoma cells CL1–5 cultured on glass, the PVC composites, polyacrylamide (PA) gels, and poly(dimethyl)siloxane (PDMS). Figure 1 demonstrates typical images of the cells cultured on the different substrates. The MTT assay quantifies metabolic active cells as the optical density (OD) at 570 nm. As shown in Fig. 2, cells grown on the glass and PA gel had the greatest viability when compared with other elastomeric substrates. The average viabilities of cells grown on the PVC composites and that of the PDMS substrates were similar. There was no significant difference in cell viability between the PVC 3∶1, 2∶1, and 1∶1. This indicates that the biocompatibility of the PVC composites is similar to that of other commonly used elastomeric substrates and not affected by varying the ratio of PVC to the plasticizer.

### Effects of substrate stiffness on filopodial length and density

**Figure 3:**
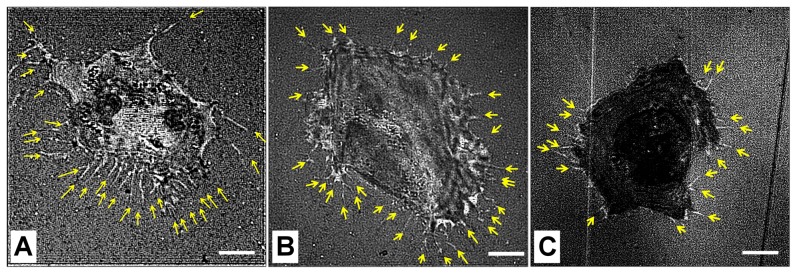
Representative SINAP images for CL1–5 cells cultured on (A) PVC 1∶1, (B) PVC 3∶1, and (C) glass substrates. Scale bar = 5 μm.

To determine whether the filopodial activities are affected by substrate stiffness, the CL1–5 cells were seeded onto the PVC 1∶1, PVC 3∶1, and glass substrates. The substrates were coated with fibronectin before cell seeding to facilitate cell attachment. Immunostaining against the coated fibronectin confirmed that the absorbed fibronectin had similar surface concentrations across the three kinds of substrates; the ratios of the mean fluorescence intensity of the stained fibronectin to the substrate background were 1.64, 1.59, and 1.41 for the PVC 1∶1, PVC 3∶1, and glass substrates respectively. The Young's moduli of the PVC composites were about 60 kPa and 20 kPa for the PVC 3∶1 and 1∶1 respectively, while the Young's modulus of glass is on the order of GPa. Live cell images were acquired using a SINAP system. Typical SINAP images for the cancer cells cultured on the PVC 1∶1, PVC 3∶1, and glass substrate are shown in Fig. 3, in which filopodia are characterized as thin, bright protrusions with various lengths at the cellular peripheries.

**Figure 4:**
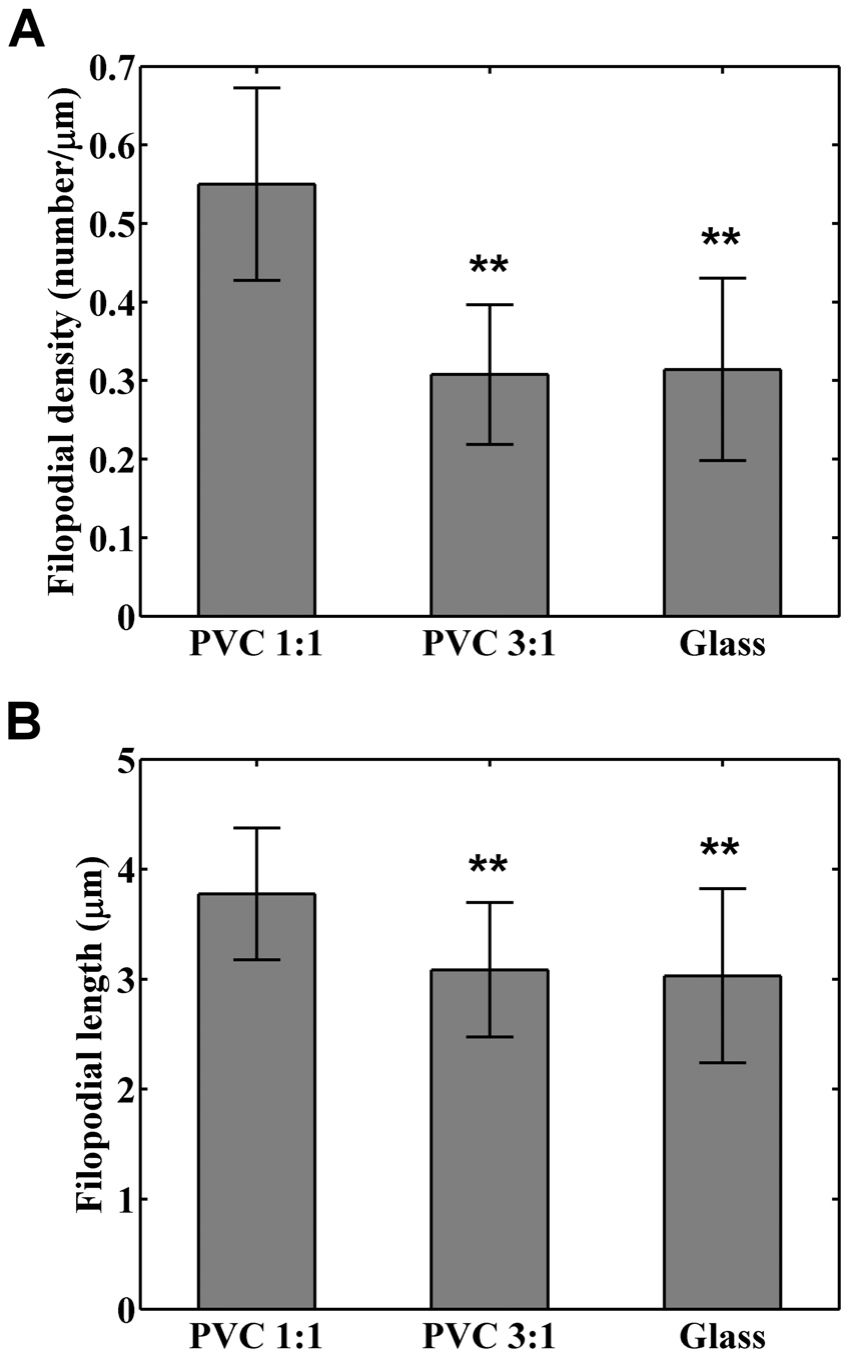
Effects of substrate stiffness on the (A) filopodial density and (B) averaged filopodial length for individual cancer cells. The filopodial density was defined as the number of filopodia per unit length of the cellular perimeter. The filopodial length was averaged from all visible filopodia of the cell. The bars represent the means of the variables and the errors denote the standard deviations calculated from 33, 18, and 43 cells for the PVC 1∶1, PVC 3∶1, and glass substrates respectively. Significant differences were found between the data of PVC 1∶1 and 3∶1, and PVC 1∶1 and glass with that ** indicates *p*<0.01.

We observed that the number of filopodia per cell ranged from 50 to 70. For individual cells, we identified all visible filopodia and calculated the density and average length of the filopodia, referred to as *f*_d_ and *f*_L_ respectively. The filopodial density was defined as the ratio of the filopodia number to the perimeter of the cell. Figure 4A shows the variation of the *f*_d_ sampled from cells cultured on PVC 1∶1, PVC 3∶1, and glass substrates, respectively. The cells cultured on the PVC 1∶1 had the largest *f*_d_ (mean  = 0.55 µm^−1^, cell number  = 33), followed by the cells cultured on the PVC 3∶1 (mean  = 0.31 µm^−1^, cell number  = 18) and that cultured on the glass (mean  = 0.31 µm^−1^, cell number  = 43). Statistical analysis revealed that there are significant difference between the results from the PVC 1∶1 and 3∶1 (*p* = 4.4×10^−9^), and the PVC 1∶1 and the glass (*p* = 1.1×10^−9^), while there is no significant difference between the results of the PVC 3∶1 and the glass (*p* = 0.75). Consistently, as shown in Fig 4B, the *f*_L_ of the cells cultured on the PVC 1∶1 was significantly longer than that of the cells cultured on the PVC 3∶1 (*p* = 8.8×10^−4^) and the glass (*p* = 2.2×10^−4^), while there is no significant difference between that of the PVC 3∶1 and the glass (*p* = 0.67). The means of *f*_L_ were 3.77, 3.08, and 3.03 μm for the PVC 1∶1, PVC 3∶1, and glass respectively. These data indicate that when cancer cells are cultured on elastomeric substrates, the cells growing on softer substrate have more and longer filopodia.

### Effects of substrate stiffness on the dynamics of filopodial extension and retraction

**Figure 5:**
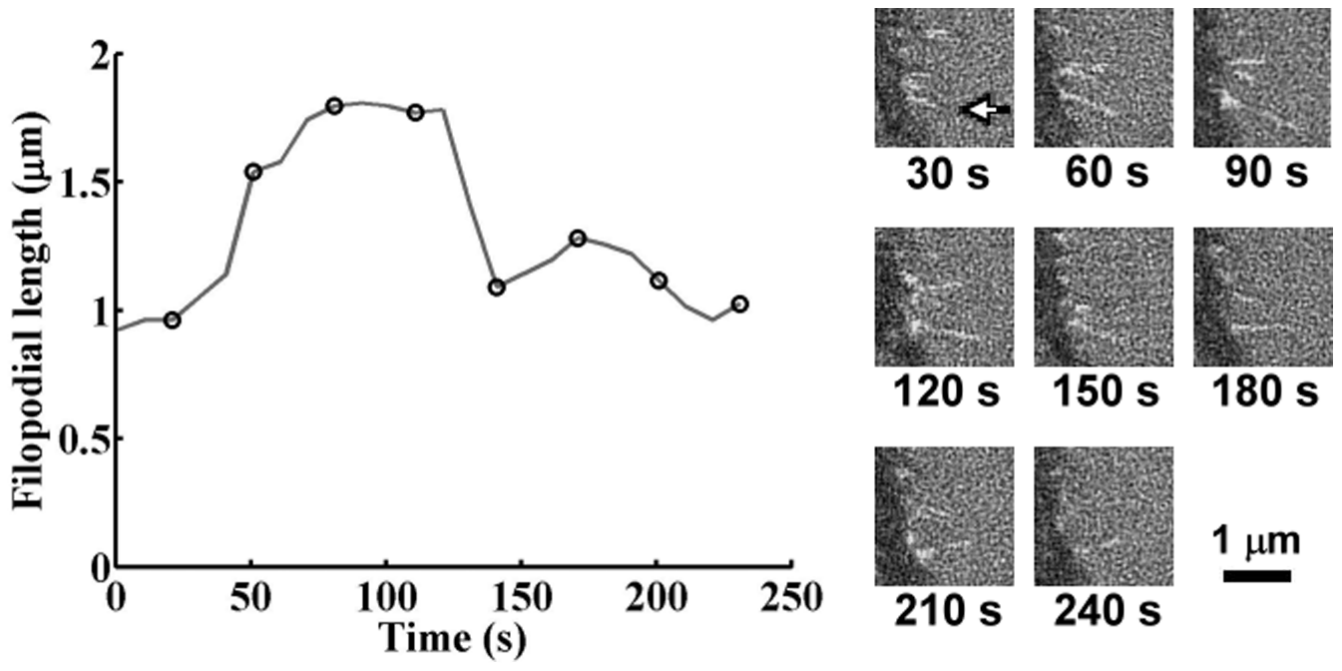
The length temporal profile of a filopodium measured from a series of SINAP images taken every 10 seconds. The cell was cultured on glass substrate. The filopodial length was 0.92 μm at the start of image acquisition, continuously extended to 1.81 μm, and retracted to 1.02 μm at the end of image acquisition. The SINAP images corresponding to the length data marked by circles are shown in the right and annotated with the recording time. The tracked filopodium was highlighted by the arrow in the 1^st^ image.

**Figure 6:**
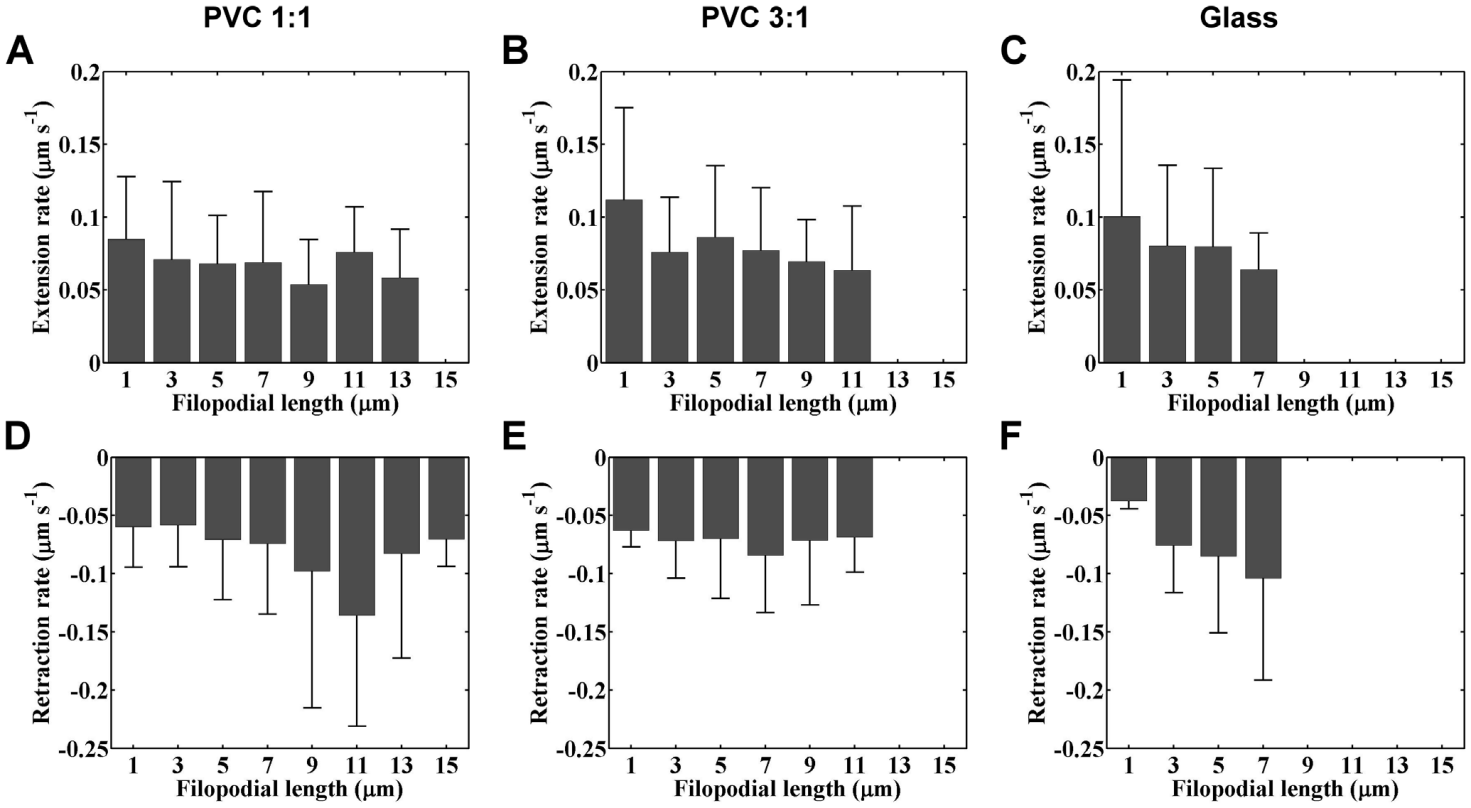
Variation of filopodial extension and retraction rate at various filopodial lengths. (A), (B), and (C) depict the relationship between the extension rates and filopodial lengths for the cells cultured on the PVC 1∶1, PVC 3∶1, and glass substrate respectively; (D), (E), and (F) show variation of the retraction rates with respect to various filopodial lengths for the cells cultured on the PVC 1∶1, PVC 3∶1, and glass substrate respectively. The bars represent the means of the rate data assorted in the length ranges and the errors denote the standard deviations. The data were assorted into a set of length ranges separated by 2 μm. The numbers of filopodia tracked for the rate measurement are 51, 59, and 34 for the PVC 1∶1, PVC 3∶1, and glass substrates respectively.

We asked if the filopodia have different rates and probabilities to extend or retract when cancer cells were cultured on substrates of various degrees of stiffness. Specifically, we reasoned that the filopodia of cancer cells cultured on stiffer substrates may have a greater propensity to retract, which leads to a smaller *f*_D_ and a shorter *f*_L_, as shown in Fig. 4. To analyze the retracting and extending dynamics of individual filopodia, time-lapsed SINAP images of live cells were taken one frame every 10 seconds for five minutes. Only filopodia lasted longer than one minute (i.e., appearing on 6 consecutive frames) were tracked and the instantaneous filopodial lengths were measured from individual frames. A representative length temporal profile of a tracked filopodium is depicted in Fig. 5. The filopodium had an initial length of 0.92 μm at start of tracking, continuously extended for 100 seconds, and retracted to 1.02 μm at the end of image acquisition. We calculated the rates of length change between consecutive frames and referred positive changes as extension and negative as retraction rates. This yielded a set of extension/retraction rates associated with various filopodial lengths. For example, the extension rates for the filopodium shown in Fig. 5 are 0.004, 0.04, and 0.001 μm⋅s^−1^ at the filopodial length of 0.92, 1.14, and 1.79 μm respectively. We tracked 51, 59, and 34 filopodia for the cells cultured on the PVC 1∶1, PVC 3∶1, and glass substrates respectively. We assumed that cells cultured on the same kind of substrates have similar filopodial dynamics and gathered together the rate data according to the type of culturing substrates. To facilitate the comparison of the rate data across various filopodial lengths, we assorted the data with a set of length ranges separated by the same gap. We first chose 2 μm as the gap; namely range 1 referred to filopodial length ≥0 and <2 µm, range 2 referred to filopodial length ≥2 μm and <4 μm, and so forth. Figure 6 summarized the variation of the rate data across the defined length ranges for cells cultured on the three kinds of substrates. The bars represent the means of the rate data assorted in the length range and the errors denote the standard deviations. In general, the extension rates exhibited a trend to decrease as the filopodial length increased, while the retraction rates appeared to increase as the length increased, with a local maximum at length of 11, 7, and 7 μm for the cells cultured on the PVC 1∶1, PVC 3∶1, and glass substrates respectively. We observed similar trends when assorting the data using different gaps such as 1 or 0.5 μm for the length ranges. Note that in Fig. 6, the rate data of the cells on softer substrates spanned larger length ranges, which is consistent with the findings shown in Fig. 4B.

**Figure 7:**
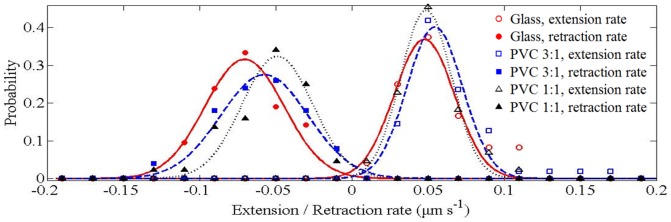
Probability distribution of the filopodial extension and retraction rates. The data were assorted into a series of rate ranges that were equally separated by 0.02 μm⋅s^−1^. The solid, dashed, and dotted lines represent normal distribution fits to the data of glass, PVC 3∶1, and PVC 1∶1 respectively. The coefficients of determination (i.e., *R-*square) for the three fittings are >0.9.

Substrate stiffness significantly affected the retraction rates when filopodial length exceeded a certain scale. We found that the significance appeared when the scale was set to be 4 μm, which is around the means of the *f*_L_ shown in Fig. 4B. For each tracked filopodium, we lumped the rate data measured at filopodial length ≥4 μm and calculated the means. We referred to the means of the extension and retraction rates as *V*_E_ and *V*_R_ respectively. We gathered together the *V*_E_ and *V*_R_ calculated from cells cultured on the same kind of substrate and assorted the data into a set of rate ranges that were equally separated by 0.02 μm⋅s^−1^. The occurring probability of a particular rate range was thus estimated by calculating the fraction of data assorted into the interested range. Figure 7 illustrates the probability distribution of the extension and retraction rates estimated for the cells cultured on the three kinds of substrates. The solid, dashed, and dotted lines represent normal distribution fits to the data of glass, PVC 3∶1, and PVC 1∶1 respectively. Statistical analysis revealed that there was no significant difference between the *V*_E_ for cells cultured on the three substrates (*p* = 0.17). The means of *V*_E_ were 0.052, 0.064, and 0.054 μm⋅s^−1^ for the cells cultured on the PVC 1∶1, PVC 3∶1, and the glass substrate respectively. However, the *V*_R_ for cells cultured on the PVC 1∶1 was significantly slower than that of the glass (*p* = 0.02), while the differences of *V*_R_ between the cells cultured on the PVC 1∶1 and the PVC 3∶1 (*p* = 0.31) and that of the PVC 3∶1 and the glass (*p* = 0.05) were not significant. The means of *V*_R_ were 0.059, 0.063, and 0.069 µm⋅s^−1^ for the cells cultured on the PVC 1∶1, PVC 3∶1, and the glass substrate respectively. These results suggest that the filopodia of cells cultured on softer substrates appear to retract slower when the filopodial length exceeds the average length.

**Figure 8:**
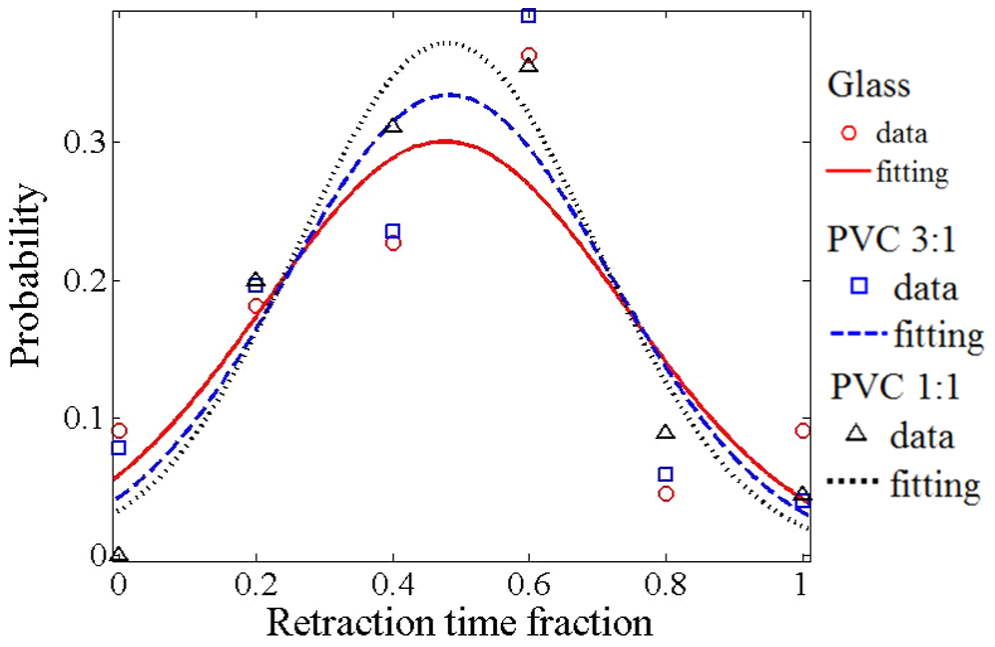
Probability distribution of the estimated tendency for a filopodium to retract. The retraction tendency was defined as the fraction of time that the filopodium spent for retraction. The solid, dashed, and dotted lines represent normal distribution fits to the data of glass, PVC 3∶1, and PVC 1∶1 respectively. The coefficients of determination (i.e., *R-*square) for the three fittings are >0.9.

To quantify the tendency of filopodial retraction when cells were cultured on substrates of particular stiffness, we defined the probability that a filopodium prefers to retract as 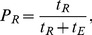
(1)where *t*_E_ and *t*_R_ denote the fractions of time that the filopodium spent for extension and retraction respectively. Since we were mainly interested in the filopodial dynamics affecting the means of the *f*_L_, only the temporal data after the filopodial length exceeding 4 μm were considered and the periods that the filopodial length remained stationary were excluded. Note that the extending and retracting probability of the filopodium are the same if *P*_R_ is equal to 0.5. Figure 8 shows the probability distribution of the *P*_R_ calculated from the cells cultured on the three types of substrates. Again, the solid, dashed, and dotted lines represent normal distribution fits to the data of glass, PVC 3∶1, and PVC 1∶1 respectively. Statistical analysis revealed that there was no significant difference between the *P*_R_ of cells cultured on the three kinds of substrate (*p* = 0.54). The means of *P*_R_ were 0.48, 0.48, and 0.47 for the PVC 1∶1, PVC 3∶1, and glass respectively. These results suggest that whether a filopodium decides to extend or retract is purely a stochastic process without dependency on substrate stiffness. Thus the variation of *f*_L_ between substrates of different degrees of stiffness primarily resulted from the varied rates of filopodial retraction, which was mainly driven by myosin II contraction and dependent on the substrate stiffness.

### Blebbistatin treatment alters filopodial activities

**Figure 9:**
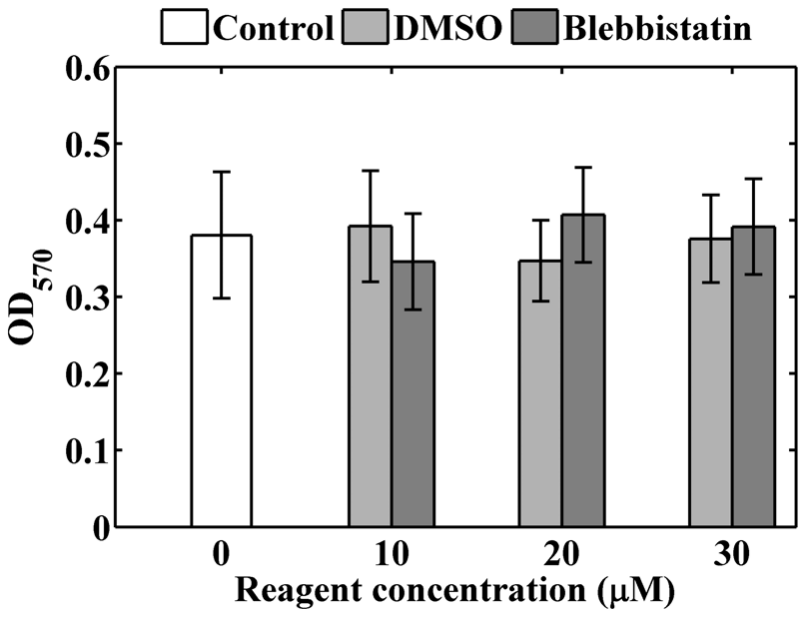
Viability of CL1–5 cancer cells with and without treatment of blebbistatin of various concentrations. The cells were cultured on glass substrates for 24μM of DMSO or blebbistatin for 1 hour (*n* = 5 for each concentration). The optical densities of cells without application of the reagents were referred to as control (*n* = 8). The bars represent the means of the variables and the errors denote the standard deviations.

**Figure 10:**
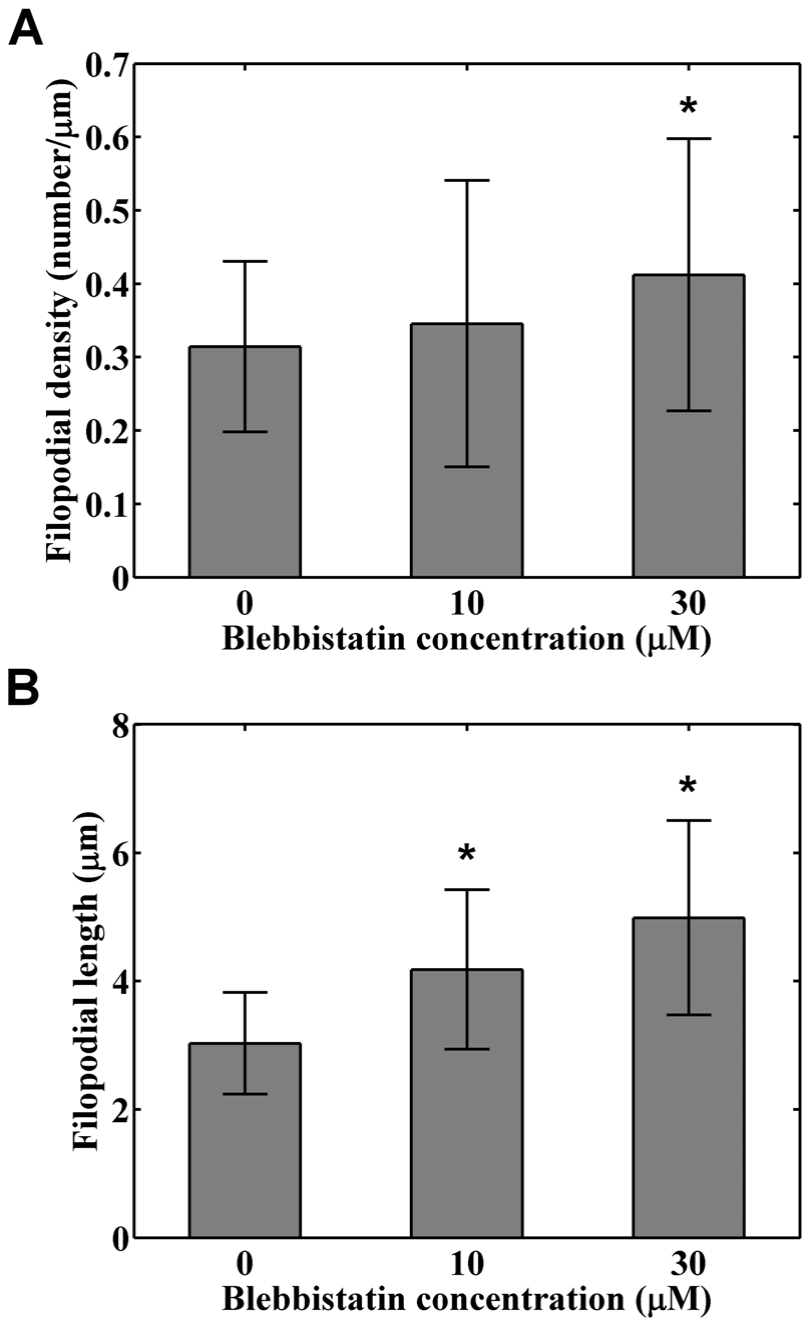
Effects of blebbistatin treatment on the (A) filopodial density and (B) averaged filopodial length for CL1–5 cells cultured on glass substrates. The bars represent the means of the variables and the errors denote the standard deviations calculated from 43, 24, and 31 cells treated with 0, 10, and 30 μM blebbistatin respectively. The mark * indicates *p*<0.05.

Since the substrate stiffness regulates the strength of cell adhesion and myosin II activities [10], we wondered whether the observed discrepancy of filopodial length and density between cells cultured on substrates of different degrees of stiffness vanishes if the myosin activities were inhibited. We first examined the viabilities and filopodial activities of cancer cells treated with blebbistatin of various concentrations. The CL1-5 cells were cultured on glass substrates for 24 hours and exposed to solutions containing 10–30 μM blebbistatin for 1 hour to inhibit myosin activities, as referring to a recent literature [24]. Cell viability studies using the MTT assay revealed that the treatment of 10–30 μM blebbistatin did not bring the CL1–5 cells significant toxicity (Fig. 9), while the filopodial density and average length increased with increasing blebbistatin concentrations (Fig. 10). Statistical analysis revealed that the filopodial density of cancer cells treated with 30 μM blebbistatin was significantly larger than that treated with 0 (*p* = 0.006) and 10 μM blebbistatin (*p* = 0.03), but there was no significant difference between that treated with 0 and 10 μM (*p* = 0.1). Likewise, the filopodial length of cancer cells treated with 30 μM blebbistatin was significantly longer than that treated with 0 (*p* = 3.2×10^−5^) and 10 μM blebbistatin (*p* = 0.02), and there was significant difference between that treated with 0 and 10 µM (*p* = 0.01).

**Figure 11:**
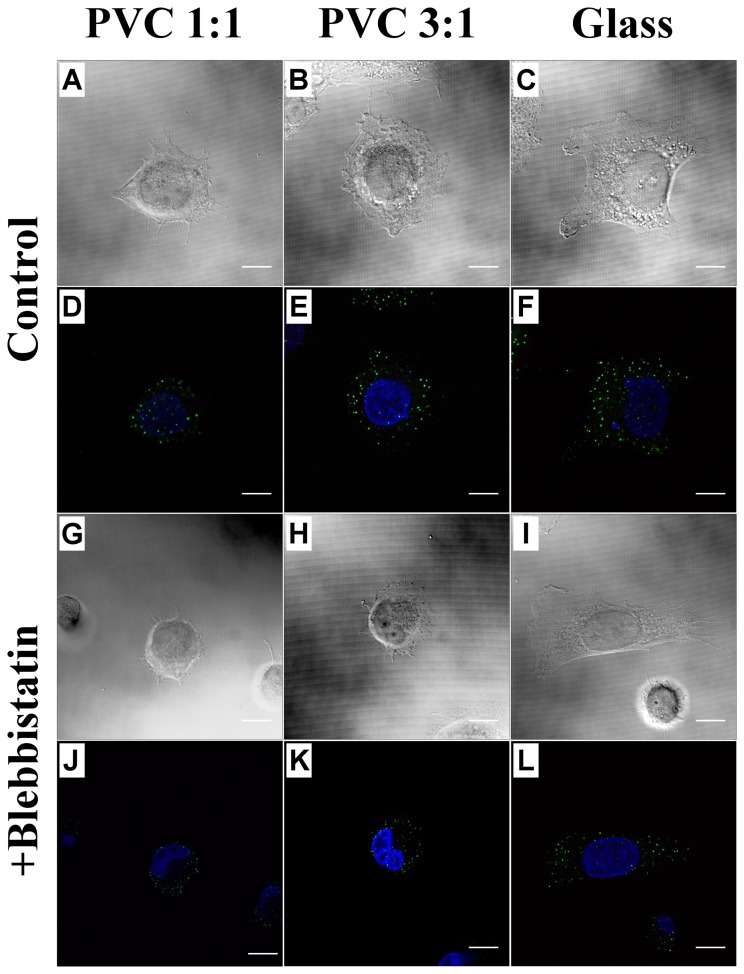
Bright field and immunofluorescence images of cells cultured on the three kinds of substrates with and without blebbistatin treatment. (A), (B), and (C) are bright field images of cells treated with regular culture medium as control; (D), (E), and (F) are their corresponding immunofluorescence images of nucleus (blue) and vinculin (green); (G)—(L) show the bright field and corresponding immunofluroresence images of cells treated with 30 µM blebbistatin for one hour to inhibit myosin II activities. Scale bar = 10 μm.

To attain significant blocking of myosin activities, we cultured the CL1-5 cells on the PVC 1∶1, PVC 3∶1, and glass substrates and treated the cells with a solution of 30 μM blebbistatin for 1 hour. Figure 11 demonstrates the representative bright field and immunostain images of cells that were treated and not treated with blebbistatin. We calculated the number of stained vinculin per cells. It appears that blebbistatin treatment increased cell rounding on all the three kinds of substrates and enhanced filopodial elongation and branching, but decreased the number of focal adhesions for cells cultured on stiffer substrates. The average and standard deviation of the vinculin number per cell were 53.2±28.3, 93.6±30.3, and 155.8±35.5 for the cells cultured on the PVC 1∶1 (*n* = 5), PVC 3∶1 (*n* = 5), and glass substrate (*n* = 8) without bleddistain treatment. After bleddistain exposure, the numbers changed to 40.5±13.6, 51.7±19.5, and 101.6±22.3 for the cells cultured on the PVC 1∶1 (*n* = 3), PVC 3∶1 (*n* = 4), and glass substrate (*n* = 9).

**Figure 12:**
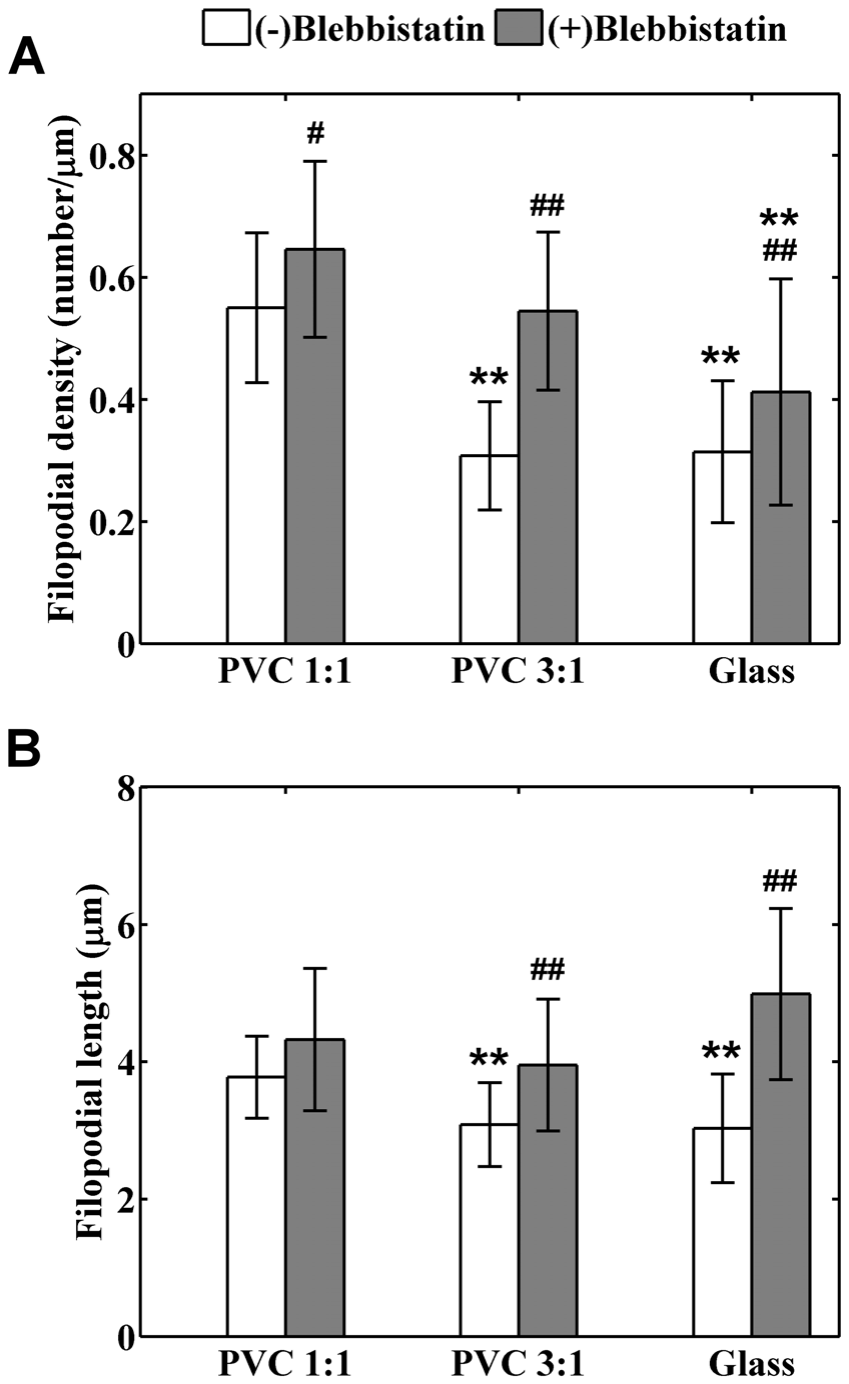
Effects of blebbistatin treatment on (A) filopodial density and (B) averaged filopodial length. The white bars represent the means of the data measured from cells without blebbistatin treatment (i.e., data in Fig. 4); the gray bars denote the means of the data measured from cells with blebbistatin treatment; and errors specify the standard deviations of the data. The numbers of cells treated with blebbistatin are 10, 13, and 31 for the PVC 1∶1, PVC 3∶1, and glass substrates respectively. # and ## denote *p*<0.05 and *p*<0.01 respectively for the difference between the data acquired from the same kind of substrates with and without blebbistatin treatment; * and ** are annotated for *p*<0.05 and *p*<0.01 respectively for the difference between the data acquired from different kind of substrates with and without blebbistatin treatment. Note that for the cells without blebbistatin treatment, significant differences were found between the data of PVC 1∶1 and PVC 3∶1, and between that of PVC 1∶1 and glass; for the cells treated with blebbistatin, significant difference was only found between the data of PVC 1∶1 and glass.

Statistical analysis revealed that after exposed to 30 μM blebbistatin for one hour, the difference of *f*_D_ between the cells cultured on the PVC 1∶1 and PVC 3∶1 became insignificant (*p* = 0.11), and the *p* value for the significance of *f*_D_ difference between that on the PVC 1∶1 and the glass decreased (*p* = 0.0069) compared with that without the blebbistatin treatment (*p* = 1.1×10^−9^). Furthermore, blebbistatin treatment significantly increased *f*_D_, with a more significant increase in cells cultured on the PVC 3∶1 (*p* = 0.046 for the PVC 1∶1, *p* = 2.1×10^−6^ for the PVC3∶1, and *p* = 0.006 for the glass). The numbers of sampled cells were 10, 13, and 31 for the PVC 1∶1, PVC 3∶1, and the glass respectively. Figure 12A summarizes the change of *f*_D_ for cells exposed to blebbistatin for one hour. Note that the results shown in Fig. 4A were plotted next to these bars for comparison.

The *f*_L_ of cells cultured on substrates of different degrees of stiffness exhibited similar change after the treatment of 30 μM blebbistatin (Fig. 12B). Again, the results shown in Fig. 4B were plotted next to those after the blebbistatin treatment for comparison. Statistical analysis revealed that the blebbistatin treatment significantly increased the *f*_L_ for cells on the PVC 3∶1 (*p* = 0.005) and the glass (*p* = 3.2×10^−5^); while the change of *f*_L_ was not significant for cells on the PVC 1∶1 (*p* = 0.48). The averages of *f*_L_ were 4.32 μm, 3.95 μm, and 4.98 μm for the PVC 1∶1, PVC 3∶1, and the glass respectively. The discrepancy of *f*_L_ between cells cultured on substrates of different degrees of stiffness became insignificant after the blebbistatin treatment (*p* = 0.12). These results suggest that the observed substrate stiffness-sensitive activities of filopodia were at least in part modulated by myosin II activity.

## Discussion

In this work, we quantified the relationship between the filopodial activities of lung cancer cells and substrate stiffness using a label-free imaging technique. We found that substrate stiffness regulated the filopodial length and density in cancer cells probably via affecting the filopodial retraction rate, which was primarily modulated by varied myosin activities. To our best knowledge, this is the first report quantitatively addressing the dependency between filopodial activities of lung cancer cells and the environmental stiffness.

The combination of the SINAP technique with the PVC-based materials allows studying the filopodial dynamics of live cells on compliant substrates without fluorescent labeling. The SINAP imaging provides sufficiently high resolution for observing single filopodium [18], [19]. However, because its signal originates from reflection, both the specimen and the substrate must have an index of refraction different from that of the culture medium. Hence most cell culturing substrates formed by hydrogels (e.g., PA gel) or siloxane-based polymers (e.g., PDMS) with refractive indices close to that of medium are not compatible with the SINAP observation. In contrast, the refractive index of PVC is close to that of glass and hence provides a superb signal enhancement in the SINAP imaging. PVC has been widely used in biomedical applications [25]. The addition of the plasticizer into PVC weakens the polymer structure and increases the flexibility of the mixture. The plasticizer used in the present study, DINCH, has a structure similar to that of *o*-phthalate, but is biodegradable and has little environmental impact and biotoxicity [26], [27]. The results of MTT assay revealed that the viability of cells grown on the PVC composites mixed with DINCH was similar to that of PDMS, although lower than that of PA gels. This may be due to that the proteins used for enhancing the cell adhesion (i.e., fibronectin) were physically adsorbed onto the surface of the PVC composites and PDMS and partially denatured due to conformation change, while the proteins were covalently conjugated to the surface of PA gels and retained most functionality.

When lung cancer cells were cultured on softer substrate, the filopodia appeared to retract at a slower rate, which promoted the development of longer and denser filopodia. The dependence between filopodial retraction rate and substrate stiffness is primarily due to varied myosin II activities [11]. The number of myosin motors engaged in contraction at a given time is thought to increase with substrate stiffening, which increases the rates of filopodial retraction [4]. Inhibition of myosin activities using blebbistatin resulted in filopodia elongation. This observation is consistent with the previous works that blebbistatin treatment decreased the retraction rate and increased the length of filopodia in neural growth cones [28], [29]. However, since there exists significant variation between cancer cells of different tissue types, whether our findings are limited to lung cancer cells or can be generalized to other types of cancer cells requires additional studies.

It is unclear why there was less significant difference between the filopodial activities of the cells cultured on the PVC 3∶1 and glass surface, and the retraction rates between the PVC 1∶1 and 3∶1. Note that the Young's moduli of the PVC composites used in this work were on the same order (i.e., 20 and 60 kPa), while the Young's modulus of the glass was on the order of GPa. Chan *et al*. reported that the rate of myosin contraction is less sensitive to the change of substrate stiffness when the stiffness exceeds a critical scale (∼1.3 kPa for neural growth cones) [11]. If this phenomenon can be generalized to other cell types, the observation that the retraction rates of the cancer cells cultured on the two PVC composites differed less significantly may be due to that the substrate stiffness is larger than the critical scale. However, it is difficult to validate whether the neuron and cancer cells have the same critical scale, since the fabrication of PVC composites softer than 1 kPa is technically challenging and may require a different plasticizer. Moreover, the filopodial dynamics were sampled at 0.1 Hz, while the filopodial retraction may be discontinuous and spent time less than the sampling interval. Thus simply dividing the change of filopodial length between two consecutive images by the sampling interval might underestimate the retraction speed. This underestimate may bias the rate distribution when the probability of faster filopodial retraction increases. Indeed, the calculated retraction rates for cells cultured on the PVC composites, about 0.06 μm⋅s^−1^, were consistent with those measured from cells cultured on substrates of similar stiffness [11], while the rates for cells cultured on glass coverslips has been reported to be around 0.16 μm⋅s^−1^ [30], roughly two times of ours. Thus the underestimate of filopodial retraction rate for cells cultured on glass may contribute to the less significance in the rate difference between the cells cultured on the PVC 3∶1 and those of the glass.

Matrix stiffness plays a critical role in regulating various tumor cell activities, such as cell growth, morphology, malignant transformation, migration, and invasiveness [4]. Together with the integrin signaling, the dynamical extending and retracting of filopodia allow tumor cells to actively probe environmental stiffness [28]. Substrate stiffness regulates filopodial length and density primarily via modulation of myosin contractility. Soft substrate promotes the development of long and dense filopodia. Long filopodia facilitate a tumor cell to spread, move and invade into the surrounding tissues [13], while higher filopodial density allows the cell to explore the environment in more directions. Our findings suggest that cancer cells surrounded by soft environment are more actively engaged in changing cellular morphology and moving directions, and searching for environmental cues for migration, while the energy used for these activities is spared when the cells stay in a stiff matrix, which probably facilitates the proceedings of other tumor-cell related activities such as growth and malignant transformation.

## Materials and Methods

### Substrates preparation

The PVC composites were prepared by mixing PVC (plastic hardener, M-F Manufacturing Corp., Fort Worth, TX, USA) with a carboxylate type plasticizer (DINCH, BASF Corp., Ludwigshafen, Germany) and the heat stabilizer ZnBa (Pau Tai Industrial Corp., Taipei, Taiwan). The stiffness of PVC composite was tuned by varying the ratio of PVC to DINCH. The mixture was gently spun, dropped onto an 18×18 mm coverslip (referred to as coverslip A), and degassed for 1 hour to expel bubbles. Another 18×18 mm coverslip, referred to as coverslip B, was grafted with a monolayer of 3-aminopropyl-trimethoxy silane (3-APTES; Sigma-Aldrich, St. Louis, MO, USA). This was done by coating the coverslip with 1 ml of 0.1 M NaOH solution for 5 min, drying the surface, spreading 0.5 ml 3-APTES solution on the NaOH-treated surface, incubating for 5 min, and rinsing the coverslip with deionized water. The coverslip B was then pressed onto the PVC mixture coated on the coverslip A, with the 3-APTES-grafted surface facing the mixture, and the mixture was cured for 15 min at 185°C in a vacuum oven (DOV-30, Deng Yng Corp., Taipei, Taiwan). The grafted 3-APTES crosslinked with PVC and prevented slippage between the PVC composite and the coverslip B [31]. The coverslip A was removed after cooling the PVC mixture back to room temperature. This yielded the PVC composite of thickness about 100 μm immobilized on the coverslip with a flattened surface.

The fabrication of a PA hydrogel-coated 18×18 mm coverslip mainly followed the protocol described by Tse and Engler [32]. Briefly, a mixture of 10% acrylamide and 0.3% bis-acrylamide solutions was prepared, and polymerized with the addition of tetramethyl-ethylenediamine and 10% ammonium persulfate. The stiffness of resulting gel was measured to be 34.9 kPa using an atomic force microscope (MFP-3D, Asylum Research, Goleta, CA, USA). The PDMS substrate was made by mixing a silicone elastomer (Sylgard 184, Dow Corning, Midland, MI, USA) with the curing agent at a 10∶1 ratio, degassed for 1 hour, spun on an 18×18 mm coverslip, and cured at 65°C for 3.5 hours, as advised by the manufacturer.

To facilitate cell adhesion, the substrate surfaces were coated with fibronectin (Sigma-Aldrich, St. Louis, MO, USA). After UV sterilization for 30 min, the PVC composites, glass coverslips, and the PDMS substrates were covered with a 50 μg/ml fibronectin in phosphate buffered saline (PBS) solution for one hour at room temperature, followed by an incubation of 4% bovine serum albumin solution (BSA; Gibco, Grand Island, NY, USA) at 4°C overnight [33]. The treatment of BSA was used to block excess coating of serum proteins onto the surface during cell culture. The PA gel was covered with a sulfosuccinimidyl-6-4-azido-2-nitrophenylamino-hexanoate solution (sulfo-SANPAH; Pierce, Rockford, IL, USA), UV activated, and incubated with a 50 µg/ml fibronectin in HEPES solution for 4 hours at 37°C, as previously described [34]. After removal of the excess protein solutions and rinsed with PBS, the substrates were immediately used for cell seeding.

### Substrate stiffness measurement

The Young's modulus of the substrate bulks was measured using a MicroTester (Instron 8848, Instron Engineering Corp., Norwood, MA, USA) following the vendor's instructions. In brief, a substrate cube with 8 mm in length and width and 4.5 mm in thickness was prepared. After attaching the bottom face of the cube to a stationary load cell, compressive forces were applied by a piston at the top face of the cube to generate static displacements with an increment of 1.75 µm (about 0.03% of the sample thickness). The sample deformations were converted to strains and plotted against the stresses of the sample measured by the load cell. The Young's modulus was then calculated from the linear-fit of the stress and strain curve between the strain range of 5% and 10%.

### Refractive index measurement

The refractive index of the PVC composites was measured with digital holographic microtomography, a novel microscopy technique capable of quantifying three-dimensional refractive index distribution of weakly scattering micrometer-scale objects such as mammalian cells. Details about the optical setup and reconstruction method have been recently described [22]. In brief, a micro trench 6 μm in depth and 10 μm in width was fabricated on a slab of the PVC composite. The trench and the composite surface were immersed in glycerol solution which served as a reference for refractive index. A laser beam with a wavelength of 405 nm was used as the light source and divided into a reference beam and a sample beam. The sample beam, after passing through the trench, was recombined with the reference beam and the resultant interference images of the trench were acquired by a camera. Two-dimensional phase images of the trench were extracted from multiple interference images with various phase shifts between the reference and the sample beams. The phase at a location was proportional to the line integral of refractive index along the path of the light ray passing through the location. In analogy to X-ray computed tomography, phase images obtained at various incident angles were used to reconstruct three-dimensional refractive index distribution of the trench based on the theory of optical diffraction tomography.

### SINAP system

The details of the working principle and setup of the SINAP system have been described previously [18], [19]. In brief, the SINAP system consisted of a standard upright microscope (Eclipse LV150, Nikon, Kanagawa, Japan) equipped with a water-immersion objective with a numerical aperture of 1.1 (CFI Plan 100×W, Nikon) and a white-light laser (Fianium, Southampton, UK) as the illumination light source. The microscope was enclosed in a heat-insulated box and kept at 37°C using a custom made heater and temperature controller during live cell imaging. A PZT-driven vertical stage (P-762.ZL, Physik Instrumente, Karlsruhe, Germany) with the smallest step size of 10 nm and a 0.1% linearity was used to control the vertical positioning of the sample. The illuminating laser beam was spread out by a diffuser, band pass-filtered,and separated into P and S waves by a polarization beam splitter. The diffuser was used to decrease the spatial coherence of the illuminating light. Note that an illuminating light with high spatial coherence corrupts the resulting images due to speckle interference. The passband of the illumination light was chosen between 550 and 750 nm to prevent from UV and heat damage on the cells. The P waves were phase-modulated by a computer-controlled, liquid-crystal spatial light modulator (SLM) (HEO 6001-SC-II, HOLOEYE Photonics, Berlin-Adlershof, Germany) that has the highest phase shifting rate of 60 Hz. A mesh pattern of illumination was generated and projected onto the sample via the focusing system of the microscope. The spatial frequency of the mesh pattern was about 2 μm^−1^ on the sample surface. This yielded a spatial resolution of 140 nm for the reconstructed image. The images were captured by a 14-bit electron-multiplying CCD camera (DU-885, Andor, Belfast, Northern Ireland) cooled down to −60°C to reduce noise. The perimeter of individual cells and the filopodia length were measured using the ImageJ (http://rsb.info.nih.gov/ij/).

### Cell line

Human lung adenocarcinoma cells CL1–5 under 30 passages were used in this study. The cells were derived from a clonal cell line CL1 as previously described [8] and kindly provided by Prof. Pan-Chyr Yang at Department of Internal Medicine, National Taiwan University Hospital and National Taiwan University College of Medicine, Taipei, Taiwan. The cells were cultured with a Dulbecco's Modified Eagle's Medium (Caisson, Logan, UT, USA) supplemented with 10% fetal bovine serum (Caisson) and 1% antibiotic (Caisson) at 37°C with a 5% CO_2_ atmosphere.

### Cell viability test

The cell viability was quantified using the MTT assay. MTT is especially useful for assaying the quantification of viable cells, because MTT is cleaved to form a formazan dye (purple color) only by metabolic active cells [20]. Cells were seeded onto the substrates prepared as aforementioned at a concentration of 10^5^ ml^−1^ and cultured at 37°C with a 5% CO_2_ atmosphere for 24 hours. The coverslips cultured with the cells were moved to new plates and treated with 1 mg/ml tetrazolium MTT (3-(4, 5-dimethylthiazolyl-2)-2, 5-diphenyltetrazolium bromide) (Bio Basic, Scarborough Ontario, Canada) for 2 hours. The resulting intracellular purple formazan was solubilized by dimethyl sulfoxide and colorimetric absorbance was quantified by measuring the optical density (OD) at 570 nm by a spectrophotometer (Tecan Group Ltd., Ma nnedorf, Switzerland).

### Cell culture chip

The live cell images were acquired using a custom-made cell culture chip mounted to the microscope. The assembly of the culture chip involved adhering a 1 mm-thick acrylic plate with a central hole of 3.5 mm in length and 2.2 mm in width onto a 10 cm culture dish, placing the substrate coverslip into the central hole, seeding the cells at a density of 10^4^ per ml, and filling the dish with culture medium. After kept at 37°C in a 5% CO_2_ atmosphere for 24 hours, the chip was mounted to the microscope for image studies and the central hole was sealed by a 24×60 mm cover glass after removal of excess medium.

### Blebbistatin treatment

To perform the myosin II inhibition experiments, the cells were first cultured with the regular medium in the chip for 24 hours, and then exposed to a culture medium supplemented with 10–30 µM blebbistatin (Sigma-Aldrich, St. Louis, MO, USA) for 1 hour before the application of MTT assay or the acquisition of SINAP images.

### Immunostaining

For fibronectin staining, the fibronectin coated surfaces were incubated with a PBS solution with 1∶400 rabbit anti-human fibronectin antibody (F3648, Sigma-Aldrich) plus 1% bovine serum albumin (BSA) at 37°C for 1 hour, and incubated with fluorophore-conjugated secondary antibodies (1∶300 Alexa Fluor 488 goat anti-rabbit antibodies in blocking solution; A-11008, Life Technologies, Grand Island, NY, USA) for 45 minutes at room temperature. For vinculin staining, cells were fixed for 10 minutes in 4% paraformaldehye (Electron Microscopy Sciences, Hatfield, PA, USA), permeabilized for minutes in 0.01% Triton X-100 (BioShop Inc., Burlington, Ontario, Canada), and blocked with 1% BSA for 1 hour at room temperature. The fixed cells were incubated with primary antibodies (1∶200 rabbit polyclonal vinculin antibody in blocking solution; Gene Tex Inc., Irvine, CA, USA) overnight at 4°C, and incubated with fluorophore-conjugated secondary antibodies (1∶250 Alexa Fluor 488 goat anti-rabbit in blocking solution; A-11008, Life Technologies) for 2 hour at room temperature. Cell nuclei were stained using 4′,6′-diamidino-2-phenylindole (DAPI, Sigma-Aldrich) dye for 10 minutes. The stained samples were mounted on slide in mounting media (DABCO, Sigma-Aldrich) and sealed with nail polish. Images were acquired using a laser scanning confocal microscope (LSM 510 Meta, or LSM 710, Zeiss, Germany) with a 100× objective lens (Plan-Apochromat Oil M27, NA 1.4, Zeiss). Series of z-stack images or single image were taken from the focal plane of staining. The Zen 2009 Light Edition software (v5.5.285.0, Zeiss) was used to adjust image intensity and contrast.

### Statistical analysis

For the data comparison between substrates of different stiffness or between different concentrations of blebbistatin, the analysis of variance (ANOVA) was employed and the Bonferroni *t*-test was performed for the mean separation process. Before the analysis, the assumption of normality was validated using Lilliefors test and the equality of variances was confirmed by the Levene's test. For the data comparison between that with and without blebbistatin treatment, either two sample unpaired *t*-test with pooled variance or Welch's approximate *t*-test was used, depending on the equality of the variances of the two samples. For this study, a *p* value less than 0.05 was considered significant.
